# Small RNA Sequencing for Profiling MicroRNAs in Long-Term Preserved Formalin-Fixed and Paraffin-Embedded Non-Small Cell Lung Cancer Tumor Specimens

**DOI:** 10.1371/journal.pone.0121521

**Published:** 2015-03-26

**Authors:** Daniel H. Buitrago, Santosh K. Patnaik, Kyuichi Kadota, Eric Kannisto, David R. Jones, Prasad S. Adusumilli

**Affiliations:** 1 Thoracic Service, Department of Surgery, Memorial Sloan Kettering Cancer Center, New York, New York, United States of America; 2 Department of Pathology, Memorial Sloan Kettering Cancer Center, New York, New York, United States of America; 3 Center of Cell Engineering, Memorial Sloan Kettering Cancer Center, New York, New York, United States of America; 4 Department of Thoracic Surgery, Roswell Park Cancer Institute, Buffalo, New York, United States of America; University of Connecticut Health Center, UNITED STATES

## Abstract

**Background:**

The preservation of microRNAs in formalin-fixed and paraffin-embedded (FFPE) tissue makes them particularly useful for biomarker studies. The utility of small RNA sequencing for microRNA expression profiling of FFPE samples has yet to be determined.

**Methods:**

Total RNA was extracted from de-paraffinized and proteinase K-treated FFPE specimens (15–20 years old) of 8 human lung adenocarcinoma tumors by affinity chromatography on silica columns. MicroRNAs in the RNA preparations were quantified by the Illumina HiSeq 2000 sequencing platform with sequencing libraries prepared with the TruSeq Small RNA Sample Preparation Kit (version 2.0) to obtain unpaired reads of 50 b for small RNA fragments. MicroRNAs were also quantified using Agilent Human miRNA (release 16.0) microarrays that can detect 1,205 mature microRNAs and by quantitative reverse transcription (RT)-PCR assays.

**Results:**

Between 9.1–16.9 million reads were obtained by small RNA sequencing of extracted RNA samples. Of these, only 0.6–2.3% (mean = 1.5%) represented microRNAs. The sequencing method detected 454–625 microRNAs/sample (mean = 550) compared with 200–349 (mean = 286) microRNAs detected by microarray. In Spearman correlation analyses, the average correlation coefficient for the 126 microRNAs detected in all samples by both methods was 0.37, and >0.5 for 63 microRNAs. In correlation analyses of the sequencing- and RT-PCR-based measurements, the coefficients were 0.19–0.95 (mean = 0.73) and >0.7, respectively, for 7 of 9 examined microRNAs. The average inter-replicate Spearman correlation coefficient for the sequencing method was 0.81.

**Conclusions:**

Small RNA sequencing can be used to obtain microRNA profiles of FFPE tissue specimens with performance characteristics similar to those of microarrays, in spite of the fragmentation of ribosomal and messenger RNAs that reduces the method's informative capacity. The accuracy of the method can conceivably be improved by increasing sequencing depth and/or depleting FFPE tissue RNAs of ribosomal RNA fragments.

## Introduction

MicroRNAs are single-stranded, non-coding RNAs of 18–25 nucleotides in length that function as epigenetic regulators by inhibiting protein translation from or inducing degradation of messenger RNA (mRNA) transcripts that they target [1, 2]. MicroRNAs are involved in crucial biological processes such as cell proliferation, differentiation, and apoptosis [3], and the dysregulation of microRNAs has been demonstrated in initiation and progression of a variety of human malignancies [4–8]. Characterization of microRNA expression has been useful in the classification, diagnosis, and prognosis of several malignancies [9–12].

Tissue specimens that have been preserved via fixation in formalin are a valuable resource for retrospective studies. Because of its simplicity and low cost, vast amounts of clinical specimens are routinely archived after formalin fixation. Unlike mRNAs, microRNAs preserve well in formalin-fixed tissue because of their ultra-short length [13, 14]; this allows the use of such tissues for microRNA measurements for clinical and research purposes. MicroRNA profiles of formalin-fixed and paraffin-embedded (FFPE) tissues are comparable to those obtained from matched, fresh-frozen tissue samples (e.g., [15, 16–19]), underscoring the suitability of FFPE specimens as appropriate resources for microRNA expression analyses. A large number of studies on the biological role as well as biomarker utility of microRNAs have consequently been performed using FFPE specimens (e.g., [20, 21]).

Hybridization microarrays are routinely used for global microRNA expression profiling of FFPE samples. Small RNA sequencing has emerged as a new technology for microRNA expression profiling. It offers the advantages of high sensitivity and specificity, detection of both novel and known microRNAs, identification of microRNA sequence polymorphisms and editing, and simpler data normalization strategies for comparing samples [22]. With the cost of adequately informative RNA sequencing assays approaching that of microarrays and with improvements to user-friendliness and robustness of methods for RNA sequencing data analysis, small RNA sequencing is likely to be used more commonly than microarrays in the near future. RNA in FFPE samples is chemically modified through crosslinking because of the formaldehyde and it is significantly fragmented [23] by oxidation from exposure to air, activity of endogenous ribonucleases during fixation, and the use of high temperatures during the paraffin-embedding process. Degradation of RNA also occurs during its isolation from FFPE samples, which involves prolonged exposure to high temperatures (55°C–70°C). Small fragments of ribosomal RNAs (rRNAs) and mRNAs that result from their degradation compete with endogenous small RNAs (e.g., microRNAs) during RNA sequencing. For instance, Sanger DNA sequencing of cDNAs cloned from FFPE tissue RNA shows that more than 80% of 18–25 nucleotide-sized small RNAs in FFPE tissue are fragments of rRNAs and transfer RNAs [24]. Despite these adverse effects of formalin fixation, it is theoretically possible to profile microRNAs in FFPE tissues if RNA sequencing is performed with an adequate depth. Therefore, we sought to examine the feasibility of small RNA sequencing for quantification of microRNAs in FFPE samples. When we initiated this work, there was only one published study that had attempted to address this question [25].

## Materials and Methods

### Ethics statement

This study was conducted with implied written and informed consent of participants and approved by the Institutional Review Board of Memorial Sloan Kettering Cancer Center (MSK) in New York, NY (IRB number WA0269–08).

### Tumor specimens

FFPE specimens of 8 therapy-naïve solitary pathologic stage I lung adenocarcinoma tumors resected at MSK between January 1995 and December 1999 were used. The 8 tumors were from a set of stage I lung adenocarcinoma cases that have been previously described [26, 27]. Rolled sections of 4 μm and 20 μm thickness were obtained from the specimens for RNA extraction. The 4 μm sections were stained with hematoxylin and eosin for histologic evaluation under a BX51 microscope (Olympus, Tokyo, Japan) to ensure that RNA was extracted from tissue with >70% tumor content.

### RNA extraction and quantification

Total RNA was isolated from 4 sections, or approximately 3 mm^3^ of each tumor, using the affinity spin column-based High Pure FFPE RNA Isolation Kit (Roche, Indianapolis, USA). RNA was quantified via absorbance spectrophotometry on a Nanodrop 2000 instrument (Thermo Scientific, Waltham, USA) and via RiboGreen dye fluorescence on a Qubit fluorometer (Life Technologies, Carlsbad, USA). Integrity of RNA was assessed using electrophoresis on Eukaryote Total RNA Pico chip on Bioanalyzer 2100 (Agilent, Santa Clara, USA). RNA aliquots were stored at -80°C.

### Microarray assay of microRNAs

Eight RNA samples were assayed in duplicate and in separate microarray experiments for the two sets of replicates. The Agilent SurePrint G3 Human miRNA 8x60k (release 16.0) microarray platform[28] was used. This platform can detect 1,205 human and 287 human viral mature microRNAs. For each of these microRNAs and control RNAs, the microarray has 1–4 different DNA probes of 40–60 b; each of these was synthesized on the microarray at 10–40 spots of 30 μm diameter. RNA (120 ng) was labeled with 3',5'-cytidine bisphosphate with cyanine dye attached to the 3' phosphate and they were hybridized in 45 μl to a microarray for 20 h at 45°C under rotation at 1/3 Hz, using reagents and methods provided with the Agilent miRNA Complete Labeling and Hybridization Kit. After post-hybridization washing, microarray slides were scanned on an Agilent G2505C microarray scanner and data from the images was extracted using Agilent Feature Extraction software (version 9.5.3). Raw microarray data was deposited in the NCBI Gene Expression Omnibus repository [29] (accession number GSE57835).

### Small RNA sequencing

One μg RNA was used to generate a small RNA sequencing library using reagents and methods provided with TruSeq Small RNA Sample Prep Kit version 2 (Illumina, San Diego, USA). Briefly, T4 RNA ligase was used to ligate RA5 and RA3 RNA oligonucleotides to 5' and 3' ends of RNA, respectively. Adapter-ligated RNA was reverse-transcribed using a RTP primer and the resulting cDNA was amplified in an 11-cycle PCR that used RP1 and indexed RP1 primers. PCR products of 140–160 bp were isolated following electrophoresis through a 6% Novex Tris-borate polyacrylamide gel (Life Technologies). Quality of the generated small RNA sequencing library was confirmed using Agilent High Sensitivity DNA Analysis Kit on Bioanalyzer 2100 instrument. Quadruplexed sequencing of libraries to generate single-end reads of 50 b was performed on Illumina HiSeq 2000 instrument using HiSeq clustering and sequencing reagents (version 2.0). Illumina HCS (version 1.4.8), RTA (version 1.12.4.2), and CASAVA (version 1.8.2) software were used for base-calling and the generation of raw, de-multiplexed sequencing data in FASTQ format. Raw sequencing data was deposited in the NCBI Sequence Read Archive [30] (accession number SRP047429).

### Reverse transcription (RT)-PCR assays of small RNAs

Briefly, 50 ng of RNA was reverse transcribed in 15 μl using a microRNA-specific oligonucleotide; reagents and methods were provided in TaqMan MicroRNA Reverse Transcription kit (Life Technologies). The cDNA (1.33 μl) was used as template in real time PCR of 40 cycles and 15 μl that was performed in triplicate on a 7900HT machine (Life Technologies) with denaturation for 15 s at 95°C and combined annealing and extension for 60 s at 60°C. SDS software (version 2.4, Life technologies) was used to calculate quantification cycle (C_q_) values with automatic baseline and threshold detection. DNA oligonucleotides provided with TaqMan MicroRNA RT-PCR assays [31] with identification numbers 397, 509, 1097, 398, 526, and 2340 (Life Technologies), were used to quantify human mature microRNAs *miR-21–5p*, -*205–5p*, -*146b-5p*, -*22–3p*, -*223–3p*, and-*423–5p*, respectively. Mature microRNAs *miR-16–5p*, -*210–3p*, and-*486–5p* were measured using custom assays that were designed based on the principle of TaqMan microRNA RT-PCR assays and were subsequently validated (references [31, 32] and S1 Table). All RT-PCR assays were performed in a single experiment and the data-set of raw C_q_ values were normalized using the global mean method [33].

### Processing and analyses of microarray data

Raw data from all microarrays were processed together using the AgiMicroRna Bioconductor package [34] (version 2.10.0) in R (version 3.0.2). Briefly, and as per optimum workflow identified for the microarray platform [35], the robust multi-chip average (RMA) method [36] was first used for probe-set summarization without background correction; the data across arrays was then normalized with the quantile method. A single outlier microarray was identified by examination of normalized data using relative log expression, unsupervised hierarchical clustering, inter-array correlation, and principal component plots; the raw microarray data was re-processed after excluding the outlier. Mature microRNA identification numbers (MIMAT IDs) of microRNAs detectable with the microarray platform were obtained from the miRBase microRNA sequence repository [37] (version 20). The value of IsGeneDetected flag, which was deermined by the Agilent Feature Extraction software, was used to determine whether microRNAs were detected by the platform.

### Processing and analyses of RNA sequencing data

Trimmomatic [38] (version 0.32) was used to trim reads of adapter and poor-quality bases with these criteria, in order: (1) remove read segments that matched sequences of adapters and primers used for sequencing library preparation; (2) remove leading/trailing bases with Phred_33_ base-quality score <3; (3) using a sliding window of 4 bases, remove the 5' terminal base if the average Phred_33_ score of the 4 bases was <15; and (4) completely discard trimmed reads with <16 remaining bases. For determining mature microRNA expression, miRExpress [39] (version 2.0) was used to map the processed reads against human pre-microRNA and mature microRNA sequences (miRBase release 20). The default settings for miRExpress was used during the process (100% identity between read and pre-microRNA sequence for alignment, read length ≥80% of mature microRNA length, and ≤4 extra leading/trailing bases in a read compared to mature microRNA sequence). To summarize count values of reads that mapped to a single mature microRNA but multiple pre-microRNAs, the rounded mean or median value of pre-microRNA-mapping counts was used when the maximum value was <50% or ≥50% of minimum, respectively. The summarized raw mature microRNA count data was then normalized across samples using the trimmed mean of M-values (TMM) normalization method, as indicated in the edgeR Bioconductor package[40] (version 3.2.4). To determine the biotypes of RNA (e.g., mRNA, rRNA, microRNA, etc.) that the sequencing reads represented, subread-align function of the Subread aligner software [41] (version 1.4.4) was used to align processed reads—with multi-mapping permitted and an ungapped genome index—to the human genome (Ensembl GRCh37.75 primary assembly). The featureCounts function [42] of Subread was then used, with multi-overlapping permitted, to identify the RNAs’ biotype that the sequencing reads represented via examination of annotations of genome locations that the reads were mapped to. The Ensembl's GRCh37.75 gene annotation data with >25 different gene_biotype attribute values was used.

### Other

For analyses that involved comparison of replicates, normalized microarray, and normalized and log_2_-transformed (with offset = 0.1) sequencing data were additionally processed with the parametric ComBat function of the SVA Bioconductor package [43] (version 3.6.0) to adjust for the batch effect of separate microarray or sequencing experiments used for replicates. For correlation analyses, normalized microarray- and sequencing-based microRNA measurements were log_2_-transformed and RT-PCR-based normalized C_q_ measurements for microRNAs were negative-transformed. Prism software (version 6.0c; GraphPad, La Jolla, USA) was used for plotting data. A *P*-value <0.05 was associated with statistical significance. RT-PCR assays and data analyses were performed at Roswell Park Cancer Institute, Buffalo, USA; all other work was completed at MSK.

## Results and Discussion

**Fig 1:**
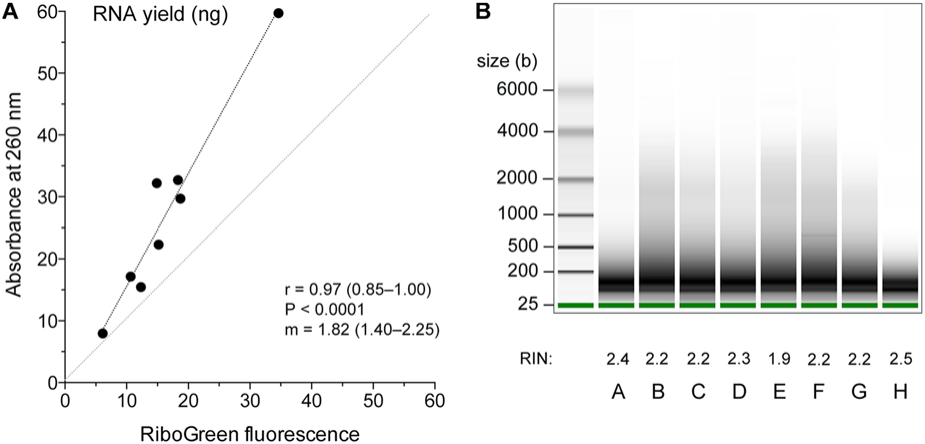
Assessment of total RNA extracted from FFPE tissues. A. Correlation between measurements of RNA yield (μg) obtained using absorbance at 260 nm or fluorescence with RiboGreen dye (n = 8). The lines of identity and linear regression (least squares method) and values and 95% confidence intervals of the Pearson coefficient (*r*) and slope of linear regression (*m*); *P*-values are also noted. B. Electrophoretogram of the 8 RNA samples (*A*-*H*). Samples were run on an Agilent Eukaryote Total RNA Pico chip on Bioanalyzer 2100. Sizes of molecular weight markers and the RNA integrity numbers are noted.

We extracted total RNA from FFPE specimens of resected stage I lung adenocarcinoma tumors. RNA was isolated using silica matrix-containing spin columns from approximately 3 mm^3^ of FFPE tissue (n = 8) after overnight digestion with proteinase K. RNA yields ranged from 8.0–59.7 μg, as quantified by absorbance at 260 nm, but 6.1–34.6 μg, as quantified with RiboGreen dye. This has more specificity for RNA than it does for DNA (Fig. 1A) and it suggests the presence of DNA in the preparations, as has been noted by others [32]. As expected, the gel electrophoresis assay on Bioanalyzer instrument showed that the RNA isolated from the FFPE specimens was highly degraded (Fig. 1B), with values of RNA integrity ranging from 1.9–2.5 [44].

Sequencing libraries were generated from 1 μg each of the total RNA samples by ligation of adapter RNAs at 5' and 3' ends, followed by reverse transcription and PCR using the Illumina TruSeq Small RNA Sample Preparation Kit. The libraries were size-selected for sequencing of RNA fragments of 15–25 nucleotides. Sequencing was performed on the Illumina HiSeq 2000 platform to obtain single-end reads of 50 bases. Between 9.1 and 16.9 million reads were obtained for each of the 8 samples (mean = 13.2 million; SD = 3.5 million; Table 1). After the reads were processed to remove nucleotide sequences of adapters used for library preparation and bases of poor quality, 67.6–%83.4% (mean = 76.8; SD = 4.5) of the reads could be mapped against the hg19 reference human genome assembly (Ensembl GRCh37.75 primary assembly). More than 90% of the mapped reads aligned against transcribed regions of the genome. However, only 0.14%–0.53% (mean = 0.29; SD = 0.13) of reads mapped to loci for microRNAs, whereas 49.4%–54.6% and 34.8%–39.3% mapped to regions that encoded for rRNAs and mRNAs, respectively (Table 1). Thus, only a very small fraction (approximately 0.1%–0.5%) of small RNAs of the 15–25 nucleotides that were isolated from the FFPE tissue specimens were microRNAs; the overwhelming majority of those were rRNA and mRNA fragments.

**Table 1:** Characteristics of small RNA sequencing data of the eight samples.

			*Median*	*Mean*	*Range*	*SD*
Total reads (10^3^)			14,039	13,220	9,095–16,936	3,474
Reads used for mapping (10^3^):			13,046	12,112	7,601–16139	3,674
	% with length 16–25 b		25.23	27.15	19.96–37.36	5.83
	% mapped by miRExpress to mature microRNAs		1.65	1.54	0.60–2.27	0.50
	% mapped by Subread to genome		77.39	76.81	67.55–83.37	4.79
	% mapped by Subread to transcribed genome:		68.08	68.63	65.16–73.51	3.37
		*RNA biotype of mapped region (%)* ^*a*^				
		MicroRNA	0.29	0.29	0.14–0.53	0.13
		Small nucleolar RNA	0.83	0.77	0.31–0.97	0.23
		Small nuclear RNA	0.42	0.43	0.17–0.76	0.17
		Long intergenic non-coding RNA	5.49	5.64	5.42–6.07	0.26
		Protein-coding RNA	36.93	37.09	34.82–39.26	1.57
		Ribosomal RNA	52.56	52.46	49.36–54.63	1.48
		Other	3.39	3.31	1.69–4.10	0.80

^a^Percentage among reads mapped by Subread to transcribed region of the genome

**Fig 2:**
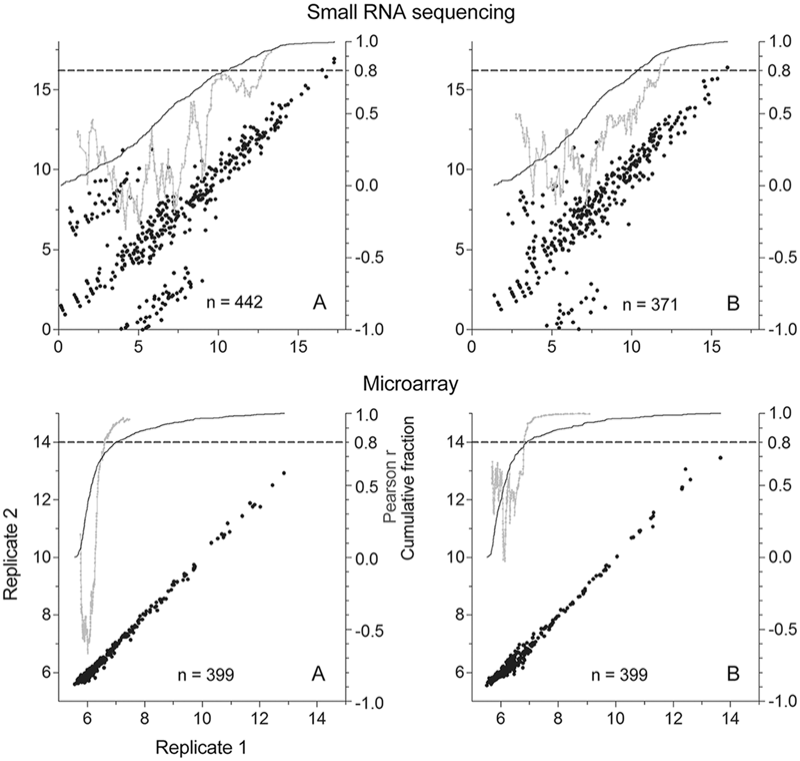
Inter-replicate correlations. MicroRNAs in 2 samples (*A* and *B*) were profiled in duplicate by both the small RNA sequencing and microarray platforms. Scatter-plots depict the inter-replicate correlation of normalized microRNA measurements. The cumulative fraction of microRNAs along the X axis (*black*), and the values of Pearson correlation coefficient of the microRNA measurements in a sliding window along the X axis of size 51 at mid-window (*gray*) are also shown.

MicroRNA expression profiles were extracted from the small RNA sequencing data using the miRExpress tool[39]. MiRExpress mapped 0.6%–2.2% of the processed sequencing reads to mature human microRNAs. Per miRBase microRNA sequence database release 20 (June 2013), 2,620 mature human microRNAs were known [37]. A total of 1,021 microRNAs were detected (mapped read count ≥1) among the 8 samples, with 454–625 detected per sample (mean = 550; SD = 69), and 283 detected in all samples. To validate the small RNA sequencing-based microRNA measurements, quantitative RT-PCR assays were used to quantify 9 arbitrarily chosen microRNAs that were considered as detected by both the microarray and small RNA sequencing platforms in all of the 8 RNA samples. In Spearman correlation analyses of the microRNA measurements obtained by RT-PCR and small RNA sequencing, the correlation coefficients were 0.19–0.95 (mean = 0.73; SD = 0.24). The coefficient value was >0.7 for 7 of the 9 microRNAs (Table 2), thereby indicating a modest accuracy of the RNA sequencing method. To estimate technical replicability of microRNA expression profiling via the sequencing method, 2 of the 8 RNA samples were subjected to small RNA sequencing in a duplicate experiment. The inter-replicate Spearman correlation coefficients for the duplicate microRNA measurements of the 2 samples were 0.75 and 0.88 (Fig. 2).

**Table 2:** Spearman correlation coefficients of normalized microRNA measurements obtained by reverse-transcription (RT)-PCR and the small RNA sequencing or microarray platforms^a^.

*MicroRNA*	*Small RNA sequencing*	*Microarray*
*miR-146b-5p*	0.83	-0.30
*miR-16–5p*	0.71	0.84
*miR-205–5p*	0.98	0.82
*miR-21–5p*	0.71	0.40
*miR-210–3p*	0.81	0.39
*miR-22–3p*	0.57	-0.36
*miR-223–3p*	0.19	0.43
*miR-423–5p*	0.79	-0.71
*miR-486–5p*	0.95	0.47

^a^Global mean-normalized C_q_ values obtained by RT-PCR assays were negative-transformed for correlation analysis

Quantification of microRNAs via hybridization of RNA to DNA probes on microarrays is currently the standard method for global microRNA profiling of FFPE tissues. To more thoroughly assess the performance of the small RNA sequencing method, we measured microRNAs in 120 ng of each of the 8 FFPE tissue RNA samples using the Agilent 8x60k Human miRNA microarray platform, which is capable of detecting 1,205 mature human microRNAs. A total of 368 microRNAs were detected (IsGeneDetected flag value = 1) among the 8 samples, with 200–349 detected per sample (mean = 286; SD = 45) and 175 detected in all samples. Of these 175 microRNAs, 126 were also detected in all 8 samples by the small RNA sequencing method. In comparisons with the RNA sequencing- and microarray-based microRNA measurements, the average The Spearman correlation coefficient for the 126 microRNAs was 0.37 and for the 63 microRNAs was >0.5. Correlation between FFPE tissue microRNA measurements obtained by small RNA sequencing and microarray platforms has been confirmed by other groups [45–47].

Microarray-based microRNA assays were replicated for 7 of the 8 RNA samples. Measured against an average inter-replicate Spearman correlation coefficient of 0.81, seen with the small RNA sequencing method, the inter-replicate correlation coefficients for the microarray platform were in the 0.98–0.99 range (mean = 0.99; SD = 0.01). However, in the Spearman correlation analyses of the microRNA measurements obtained by RT-PCR and microarray assays, the correlation coefficients were -0.36–0.84 (mean = 0.22; SD = 0.55) and were smaller than those observed in the RNA sequencing-based measurements (Table 2). Thus, whereas the small RNA sequencing platform detected a greater quantity of microRNAs with greater accuracy than the microarray platform, its precision was not as good. Table 3 summarizes the performance characteristics that were observed in the 2 microRNA profiling platforms used in this report.

**Table 3:** Comparison of the small RNA sequencing and microarray platforms.

	*Small RNA sequencing*	*Microarray*
Detection of microRNAs		
Platform capability	2,620^*a*^	1,205
Detected in any sample	1,021 (39.0%)	368 (30.5%)
Detected in all 8 samples	283 (10.8%)	175 (8.5%)
Detected per sample (mean, range, SD)	550, 454–625, 69	286, 200–349, 45
Inter-replicate Spearman correlation^*b*^ (mean, range, SD)	0.81, 0.75–0.88, 0.09 (n = 2)	0.99, 0.98–0.99, 0.01 (n = 7)
Spearman correlation with RT-PCR measurements^*c*^ (mean, range, SD; n = 9)	0.73, 0.19–0.98, 0.24	0.22, -0.71–0.84, 0.55

^a^Known human mature microRNAs in miRBase release 20

^b^Coefficient value of Spearman correlation analyses

^c^Global mean-normalized C_q_ values were negative-transformed for correlation analysis

Overall, our findings demonstrate the feasibility of small RNA sequencing for microRNA profiling of FFPE tissue RNA in spite of its compromised quality and integrity. Even though only 1.54% of 12.1 million small RNA sequencing reads of FFPE tissues were identified as representing 550 known microRNAs in this study, the number of microRNAs that were detected and quantified was more than that by the microarray platform (Tables 1 and 3). The Spearman correlation coefficients for measurements obtained by the sequencing and microarray platforms for half of the 126 microRNAs that were detected in all samples by both platforms was >0.5. In correlation analyses of the sequencing- and RT-PCR-based measurements of nine microRNAs that were examined, the coefficients were >0.7 for 7 (Table 2). The technical replicability of the small RNA sequencing method was, however, relatively poor compared to the microarray method (average inter-replicate Spearman correlation coefficients of 0.81 and 0.99, respectively; Table 3). It is conceivable that the precision of the sequencing method could be improved by increasing the sequencing depth to obtain a greater number of reads and by utilizing methods to deplete rRNA from the RNA preparations [48–50] or to reduce its representation in the sequencing library using strategies such as 'poison primer' blocking[24].

Only one study [25] had reported that the feasibility of using small RNA sequencing for microRNA profiling of FFPE tissue RNA when we initiated the work described in this paper. Since then within the past year, at least 5 other studies have shown that RNA sequencing can be used to accurately profile microRNAs in FFPE samples (S2 Table) [45–47, 51, 52]. These studies mostly performed from cell lines and human FFPE tissues of 2–9 years old have shown that microRNA expression profiles obtained by small RNA sequencing are highly concordant between FFPE and fresh-frozen tissues [25, 47, 51, 52], and that small RNA sequencing and microarray platforms generate similar microRNA profiles of FFPE tissues [25, 47]. The findings of these and our studies performed on NSCLC specimens of 15 to 20 years old therefore demonstrate that small RNA sequencing can be used to obtain microRNA profiles of FFPE tissue specimens with performance characteristics similar to those of microarrays.

## Supporting Information

S1 Table Custom TaqMan microRNA reverse transcription (RT)-PCR assays for human mature microRNAs *miR-210–3p* and *miR-486–5p*. (DOCX)

S2 Table Six studies that have assessed RNA sequencing for profiling microRNAs in formalin-fixed, paraffin-embedded tissues. (DOCX)
