# Supplementary material for: Small RNA Sequencing for Profiling MicroRNAs in Long-Term Preserved Formalin-Fixed and Paraffin-Embedded Non-Small Cell Lung Cancer Tumor Specimens
Source: PLoS One. 2015 Mar 26;10(3):e0121521. doi: 10.1371/journal.pone.0121521 (PMC4374839; doi:10.1371/journal.pone.0121521)
Supplement: S1 Table — (DOCX) [file pone.0121521.s001.docx]

**Table S1.** Custom TaqMan™ microRNA reverse-transcription (RT)-PCR assays^a^ for human mature microRNAs *miR-210-3p*, *miR-372-5p* and *miR-486-5p*

|  | *miR-210-3p* | *miR-486-5p* |
| --- | --- | --- |
| Analyte | cugugcgugugacagcggcuga | uccuguacugagcugccccgag |
| RT primer | gtcgtatccagtgcagggtccgaggtatt-cgcactggatacgactcagcc | gtcgtatccagtgcagggtccgaggt-attcgcactggatacgactcggggc |
| 5' PCR primer | cgcgcctgtgcgtgtgacag | cgcgctcctgtactgagc |
| 3' PCR primer | gtgcagggtccgaggt | gtgcagggtccgaggt |
| TaqMan™ probe^b^ | atacgactcagccgct | atacgactcggggcagct |

^a^Concentrations of RT and PCR primers, and TaqMan probes in reactions were 50 nM, 1 µM and 100 nM respectively. Validity of the assays was confirmed by assessment of dynamic range, comparison against TaqMan microRNA RT-PCR assays from Life Technologies for quantification cycle (C_q_) values, lack of a signal in the absence of reverse transcriptase, and generation of a single PCR product (data not shown).

^b^With a 5' *6*-carboxyfluorescein fluorophore and a 3' TAMRA quencher with a minor goove binder molecule attached to it
