# Supplementary material for: Small RNA Sequencing for Profiling MicroRNAs in Long-Term Preserved Formalin-Fixed and Paraffin-Embedded Non-Small Cell Lung Cancer Tumor Specimens
Source: PLoS One. 2015 Mar 26;10(3):e0121521. doi: 10.1371/journal.pone.0121521 (PMC4374839; doi:10.1371/journal.pone.0121521)
Supplement: S2 Table — (DOCX) [file pone.0121521.s002.docx]

**Table S2.** Six studies that have assessed RNA sequencing for profiling microRNAs in formalin-fixed, paraffin-embedded tissues

|  | *Weng 2010* | *Kelly 2013* | *Kolbert 2013* | *Meng 2013* | *Plieskatt 2014* | *Tam 2014* |
| --- | --- | --- | --- | --- | --- | --- |
| Sample-size | 6 | 5 | 6 | 8 | 5 | 3 |
| RNA isolation | Ambion RecoverAll | Qiagen RNeasy | Ambion RecoverAll | Ambion RecoverAll | Qiagen miRNeasy | Norgen Biotek FFPE |
| Library preparation | Illumina TruSeq Small RNA, v1.5 | Illumina TruSeq Small RNA, v 1.5 | Illumina TruSeq Small RNA, v 1.5 | Life Technologies SOLiD Small RNA | Illumina TruSeq Small RNA | Illumina TruSeq Small RNA |
| Ribosomal RNA depletion | No | No | No | No | Epicentre Ribo-Zero | No |
| Sequencer | Illumina GAII | Illumina GAIIx | Illumina GAIIx/HiSeq | Life Technologies SOLiD | Illumina GAIIx | Illumina HiSeq 2000 |
| Total raw reads (million) | 10-12 | 24-44 | Unknown | 10-12 | 25-36 | Unknown |
| Read alignment software | Novocraft Novoalign | miRExpress 2.0 | Illumina ELAND | Custom code | miRDeep 2.0 and miRExpress 2.0 | Novocraft Novoalign |
| Reads from microRNAs (%) | 43-58 | 6-17 | 70-78 | 20-85 | 63 | Unknown |
| Conclusion | Similar microRNA profiles of FFPE and frozen tissues | Moderate correlation with Illumina DASL bead microarray-based microRNA quantification | Moderate correlation with microRNA measurements obtained using NanoString nCounter technology, or Agilent 2.0 or Affymetrix miRNA 1.0 microarrays | Similar microRNA profiles of FFPE and frozen tissues | Moderate correlation with Agilent 16.0 microarray-based microRNA quantification | Similar microRNA profiles of FFPE and frozen tissues |
